# A Comparative Study of Operative Versus Non-operative Management of High-Grade Splenic Injuries in a Tertiary Care Center in India

**DOI:** 10.7759/cureus.50010

**Published:** 2023-12-05

**Authors:** Pradeep Chandran, Alex Chakiath, Sulfekar Meera Sainaba, Prashant Girijavallabhan Nair, Jayas Siby, Gowri Madhusudanan Pillai, Amjad Ali Khan, Alfin Jose, Deepak Pradeep Kumar, Jerin George Viju

**Affiliations:** 1 Trauma Surgery, King's College Hospital, London, GBR; 2 Surgical Oncology, Malabar Cancer Centre, Thalassery, IND; 3 General Surgery, Government Medical College, Thiruvananthapuram, Thiruvananthapuram, IND; 4 General Surgery, Wirral University Teaching Hospital, Liverpool, GBR; 5 General Surgery, Whipps Cross Hospital, London, GBR; 6 General Surgery, West Middlesex University Hospital, London, GBR; 7 General Surgery, Daya General Hospital, Thrissur, IND; 8 General Surgery, Government Medical College, Thrissur, Thrissur, IND

**Keywords:** haemoperitoneum, blunt abdomen trauma, nom, blunt abdominal injury, splenic trauma

## Abstract

Background

The spleen is one of the most common solid organs injured in blunt abdominal trauma with significant mortality. The management of splenic injury has significantly changed over the last few decades, ranging from certain splenectomies to non-operative management (NOM). Although several retrospective studies have been published on the NOM of minor spleen injuries, few studies have analyzed the results of NOM for high-grade splenic injuries. The pertinent question that we attempt to answer is, "Is it possible to manage extensive splenic injuries non-operatively?".

Objectives

To study the feasibility of NOM for the American Association for the Surgery of Trauma (AAST) Grade 3, 4, and 5 splenic injuries and to assess the demographic profile and cases for AAST Grade 3, 4, and 5 splenic injuries.

Methods and methodology

We, retrospectively, studied patients admitted with AAST Grade 3, 4, and 5 splenic injuries from blunt abdominal trauma admitted at the Government Medical College, Thiruvananthapuram, India, between January 2014 and October 2020. Their demographics, grade of splenic injuries, associated injuries, and methods of management were collected, and statistical analysis was done.

Results

The study included 132 patients with AAST Grade 3, 4, and 5 splenic injuries. Fifty percent of patients had Grade 3 injuries, 39.4% had Grade 4 injuries, and 10.6% were found to have Grade 5 splenic injuries. Grade 3 and 4 injuries were mainly managed non-operatively, while Grade 5 injuries had a failure rate of nearly 65% when managed non-operatively. Additionally, 73.5% of splenic injuries were successfully managed non-operatively. A significant association was noted between the severity of injuries and the need for operative management (p<0.001). Meanwhile, 64.29% of the patients with Grade 5 splenic injuries ended up needing operative management, as opposed to 34.62% in Grade 4 and 12.12% in Grade 3 splenic injuries.

Conclusion

We suggest that NOM may be undertaken successfully in appropriately designed areas with close observation for hemodynamically stable patients with extra vigilance in the case of the elderly and those with associated injuries. There should be a low threshold for switching to operative management, especially in Grade 5 injuries.

## Introduction

Throughout history, the splenic worth has vacillated from the lofty pedestal of an essential organ for life to the lowly depth of a vestigial organ without merit [1]. The role of the spleen in combating infection has been considered for many years. The first such report was given by King et al. in 1952 [2]. Although Bilroth reported in an 1881 autopsy that splenic injury might have healed spontaneously, Hamilton Bailey, as recently as 1972, asserted that “surgery is always needed.” Though a dictum of mandatory splenectomy in splenic injury existed until the 1970s [3-5], it was noticed that splenectomy increased the risk of infections with its most deadly manifestation, overwhelming post-splenectomy infection (OPSI), reported between 1950 and 1970 [6]. It was in the 1980s that there was widespread recognition of the immune functions of the spleen [6,7]. Interestingly, the current non-operative paradigm in adults was stimulated by the success in managing hemodynamically stable pediatric patients. Non-operative management (NOM) is not 100 percent safe and is deemed to fail in 2-33% of cases [8]. NOM failures have been reported in up to 33% of Grade 4 and 75% of Grade 5 splenic injuries [8]. Grade of injury, presence of other intra- and extra-abdominal injury, age >55 years, and neurological status of the patient were all considered causes of failure [8]. Physiological deterioration has been reported as the most important deciding factor in operative management, with 0.7-26.5% due to accompanying hollow viscous injury [8].

## Materials and methods

This was a retrospective study done in a tertiary trauma care center in South India. The patient records from January 1, 2014, to October 10, 2020, were perused. Patients who had American Association for the Surgery of Trauma (AAST) Grade 3, 4, and 5 splenic injuries following blunt trauma to the abdomen, proved by computed tomography (CT) imaging, and patients above the age of 13 years were included in the study. Their age, mode of injury, treatment plan, and failure of NOM were recorded in a Microsoft Excel spreadsheet. Statistical analysis was done using Pearson's chi-square test to determine if the findings were significant. A p value of <0.05 was considered to be statistically significant.

Patients who presented to the emergency department following trauma were evaluated with history and clinical examinations with primary and secondary surveys in trauma. As an extension of clinical examination, focused assessment with sonography for trauma (FAST) was performed. A CT scan of the abdomen and pelvis was done in FAST-positive patients. Patients with AAST Grade 3, 4, and 5 splenic injuries were included in the study. All preoperative investigations were sent at the time of admission, and patients were admitted to either a high-dependency care unit or an intensive care unit for close monitoring.

Those patients who were hemodynamically unstable underwent exploratory laparotomy and splenectomy. Hemodynamically stable patients were managed with NOM. The NOM protocol followed was the following:

(1) Monitoring of vitals, including pulse rate, blood pressure, and respiratory rate, was done hourly. (2) Clinical examination was every four hours. (3) Evaluation of hematocrit every four hours for the first two days and then 12 hours till the day of discharge. (4) Strict bed rest for the initial two days. Light mobilization was permitted from day three. Laxatives and antitussives were given to avoid undue straining.

Failure on NOM was considered when:

(1) Persistent tachycardia of more than 130 beats per minute (2) Persistent hypotension of systolic blood pressure less than 90 mm of Hg or (3) A fall in hemoglobin, a rate of fall of 2% within two consecutive hemoglobin values, four hours apart, in spite of at least two units of packed red cells.

## Results

**Figure 1:**
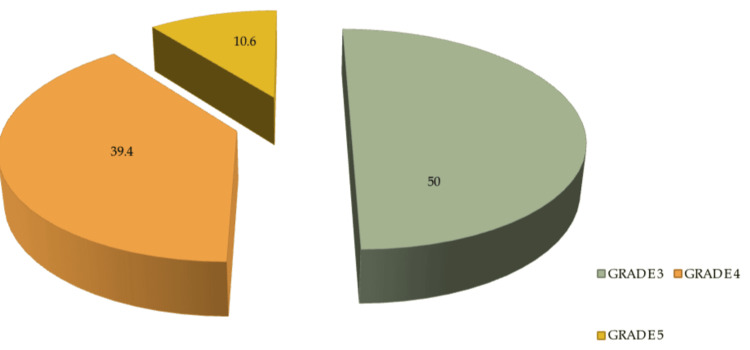
Percentage of high-grade splenic injuries

The study population was found to be n=132 patients with AAST Grade 3, 4, and 5 splenic injuries. Fifty percent (n=66) of patients had Grade 3 injuries, 39.4% (n=52) had Grade 4 injuries, and 10.6% (n=14) had Grade 5 splenic injuries, as shown in Figure 1.

**Figure 2:**
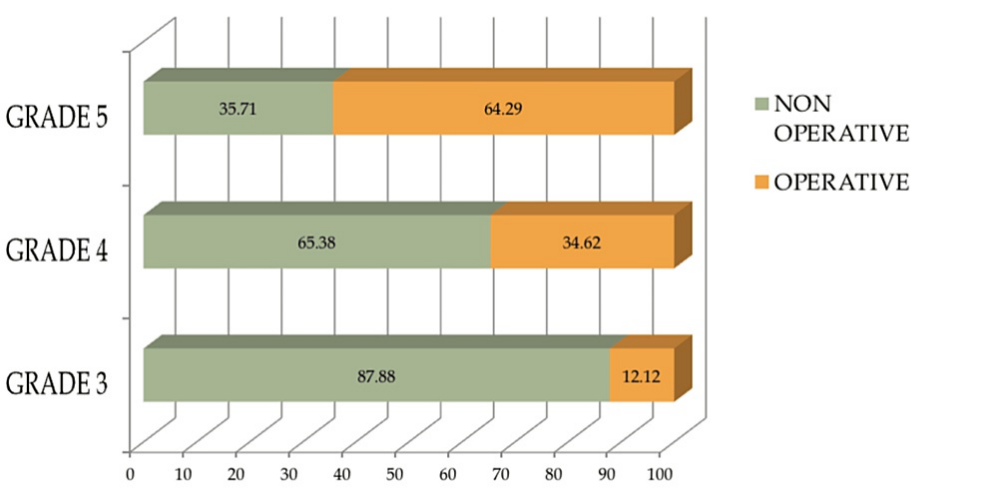
Percentage of NOM vs operative management in high-grade splenic injuries

Additionally, 73.5% (n=97) of splenic injuries were successfully managed non-operatively. A significant association was noted between the severity of injuries and the need for operative management (p<0.001). Specifically, 64.29% (n=9) of the patients with Grade 5 splenic injuries ended up needing operative management, as opposed to 34.62% (n=18) in Grade 4 and 12.12% (n=8) in Grade 3 splenic injuries, as illustrated in Figure 2.

There was no association between the mode of treatment and duration of hospital stay with a median length of stay of 10.2 days (p=0.544). Splenic injuries were predominantly seen in males (86.4%, n=114). Age was not found to be a contraindication for NOM and had no association with failure rates. The median age of patients was found to be 42.5 years. The most common cause of splenic injuries was found to be road traffic accidents (62.1%, n=82), followed by fall from height (21.2%, n=28).

**Figure 3:**
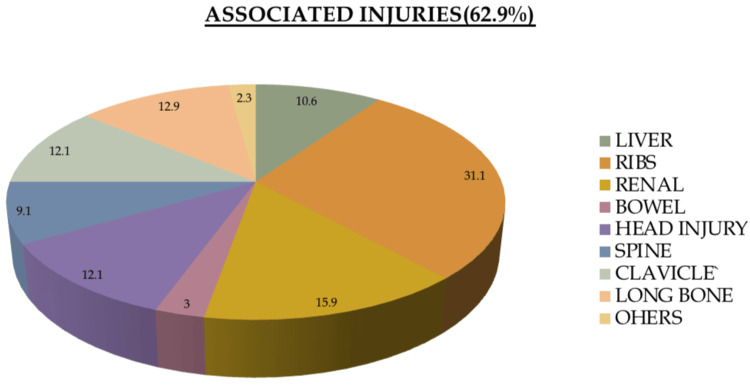
Associated injuries

Additionally, 62.9% (n=83) of the patients had other associated injuries, 31.1% (n=41) had rib fractures, 15.9% (n=21) had renal injuries, 12.1% (n=16) had head injuries, and 10.6% (n=14) had liver injuries. Blunt trauma to the left chest was the most common associated injury, and hollow viscous injury was found in 3% (n=4) of cases, as shown in Figure 3. Bowel injuries posed a significant threat to NOM (p=0.026). There were no in-hospital mortalities or incidences of re-bleed. The most common complications associated with splenic injuries were found to be respiratory infections in 4.54% (n=60) of patients.

## Discussion

The liver and spleen are the most common intra-abdominal solid organs susceptible to blunt trauma injury. NOM of splenic injuries had almost 100% mortality in the early 1900s. Hence, splenectomy was the treatment of choice for splenic injuries [1]. In 1952, King et al. identified that splenectomies done in children led to the subsequent development of severe fatal infections [2]. They concluded that the spleen, being a reservoir of lymphocytes, results in an increase in the susceptibility of the child to develop severe infections, if removed. Post splenectomy, the immune response to intravenous antigens is noted to be less in terms of decreased formation of IgM, decreased phagocytosis-stimulating proteins such as tuftsin, and decreased intravascular clearance of particles [6]. The delay in immune response in the absence of a spleen leads to decreased antibody production against encapsulated bacteria such as Streptococcus pneumonia (50%-90%), Neisseria meningitides, Haemophilus influenzae, and Streptococcus pyogenes (25%), leading to OPSI, which is rare, but rapidly fatal [7].

Until the 1970s, operative management of splenic injuries was preferred. In the 1980s, reports on NOM for splenic injury came to light in the pediatric population. This was attributed to the higher proportion of myoepithelial cells in the spleens of children, with more efficient contraction and retraction of the splenic arterioles. A thicker splenic capsule and more elastic rib cages in children prevent transmission of the direct force of the trauma to the spleen [8]. Several reasons prohibited clinicians from bravely venturing into NOM in adults. The structural and vascular changes in the spleen according to age, type of trauma, and force of trauma were thought to make spontaneous hemostasis unlikely [9].

Lucas noted in an autopsy study that the spleen has the ability to self-heal [3]. Additionally, blunt injuries of the spleen occur perpendicular to the organ’s axis, which decreases the risk of segmental vascular damage [3]. CT imaging provides an accurate assessment of the grade of a splenic injury and other associated injuries, such as pneumothorax, hollow viscous injuries, and injuries in the retroperitoneum, thereby helping choose an ideal treatment plan [4]. Most spleen injuries show a healing pattern at follow-up imaging after three to six months [3,4]. The CT scoring of a splenic injury given by Kozar et al. [10] and adopted by the AAST is shown in Table 1.

**Table 1:** American Association for the Surgery of Trauma (AAST) grading of splenic injuries As described by Kozar et al. [10].

Grade	Injury	Description
1	Laceration	Capsular tear, <1 cm parenchymal depth
2	Haematoma	Subcapsular, 10-50%, surface area
		Intraparenchymal, <5 cm in diameter
	Laceration	1-3 cm Parenchymal depth, which does not involve a trabecular vessel
3	Haematoma	Subcapsular, >50% surface area or expanding; ruptured Subcapsular or parenchymal haematoma
		Intraparenchymal haematoma >5 cm or expanding
	Laceration	>3 cm parenchymal depth or involving trabecular vessels
4	Laceration	Laceration involving segmental or Hilar vessels producing major devascularization (25% of the spleen)
5	Laceration	Completely shattered spleen
	Vascular	Hilar vascular injury that devascularizes the spleen

Irrespective of the grade of injury, any hemodynamic instability is an absolute indication of emergency laparotomy. NOM could be attempted in patients less than 55 years old who are hemodynamically stable, are conscious, lack signs of peritonitis on examination, and do not require transfusions of more than four units [11].

NOM includes observation and angioembolization with a low threshold for splenectomy. All AAST Grade 3 or more splenic injuries are recommended to undergo angiography with embolization as it increases the salvage rates in the spleen. However, little is known about the definitive amount of the spleen required for optimum functioning of the spleen. Additionally, high-grade injuries may fail NOM with angioembolization, leading to delayed splenectomy [12].

We reported a success rate of 73.5% for NOM, though there was a high failure rate among Grade 5 injuries, which is comparable to the 76.9% success rate reported by NIjdam et al. [13], underscoring the viability of NOM in high-grade injuries. Boyuk et al. reported that the grade of a splenic injury was a significant predictor for the success of NOM, which aligns with our observation of higher operative management needs in higher-grade injuries [14]. Ali et al. also emphasized the importance of hemodynamically stability and injury grade in management decisions [15].

The main limitation of this study is that the impact of angioembolization has not been studied, as the facility for angioembolization was not present at the center where the study was conducted, though Nann et al. found the effectiveness of embolization, as opposed to observation alone [16].

## Conclusions

We recommend that NOM may be undertaken in high-grade splenic injuries in patients who are hemodynamically stable, under close observation in appropriate high-dependency units, with monitoring of hemoglobin and vital parameters. There should be a low threshold to switch to operative management. NOM has the advantage of preventing morbidity and mortality of infective complications in patients post splenectomy, particularly OPSI.
